# Microcontact-Imprinted Optical Sensors for Virulence Factors of Periodontal Disease

**DOI:** 10.1021/acsomega.3c00389

**Published:** 2023-04-19

**Authors:** Thomas Hix-Janssens, Sudhirkumar Shinde, Rahma Abouhany, Julia Davies, Jessica Neilands, Gunnel Svensäter, Börje Sellergren

**Affiliations:** † Department of Biomedical Science, Faculty of Health and Society, Malmö University, 205 06 Malmö, Sweden; § Section for Oral Biology and Pathology, Faculty of Odontology, Malmö University, 205 06 Malmö, Sweden

## Abstract

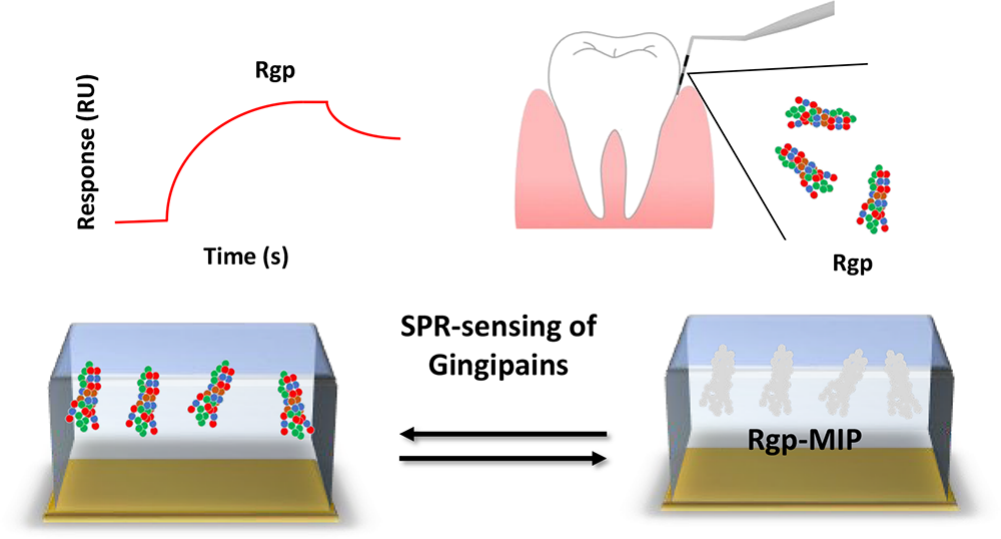

Periodontitis (gum disease) is a common biofilm-mediated oral condition, with around 7% of the adult population suffering from severe disease with risk for tooth loss. Moreover, periodontitis virulence markers have been found in atherosclerotic plaque and brain tissue, suggesting a link to cardiovascular and Alzheimer’s diseases. The lack of accurate, fast, and sensitive clinical methods to identify patients at risk leads, on the one hand, to patients being undiagnosed until the onset of severe disease and, on the other hand, to overtreatment of individuals with mild disease, diverting resources from those patients most in need. The periodontitis-associated bacterium, *Porphyromonas gingivalis*, secrete gingipains which are highly active proteases recognized as key virulence factors during disease progression. This makes them interesting candidates as predictive biomarkers, but currently, there are no methods in clinical use for monitoring them. Quantifying the levels or proteolytic activity of gingipains in the periodontal pocket surrounding the teeth could enable early-stage disease diagnosis. Here, we report on a monitoring approach based on high-affinity microcontact imprinted polymer-based receptors for the Arg and Lys specific gingipains Rgp and Kgp and their combination with surface plasmon resonance (SPR)-based biosensor technology for quantifying gingipain levels in biofluids and patient samples. Therefore, Rgp and Kgp were immobilized on glass coverslips followed by microcontact imprinting of poly-acrylamide based films anchored to gold sensor chips. The monomers selected were N-isopropyl acrylamide (NIPAM), N-hydroxyethyl acrylamide (HEAA) and N-methacryloyl-4-aminobenzamidine hydrochloride (BAM), with *N*,*N*′-methylene bis(acrylamide) (BIS) as the crosslinker. This resulted in imprinted surfaces exhibiting selectivity towards their templates high affinity and selectivity for the templated proteins with dissociation constants (*K*_d_) of 159 and 299 nM for the Rgp- and Kgp-imprinted, surfaces respectively. The former surface displayed even higher affinity (*K*_d_ = 71 nM) when tested in dilute cell culture supernatants. Calculated limits of detection for the sensors were 110 and 90 nM corresponding to levels below clinically relevant concentrations.

## Introduction

Periodontitis is a common biofilm-mediated oral condition, with around 7% of the adult population suffering from severe disease and tooth loss.^1,2^ Accurate diagnostic methods to identify patients at risk of tooth loss are lacking, explaining the tendency of overtreatment of individuals with mild disease. At the moment, the four main methods to diagnose periodontitis by dentists are inspection, palpation, probing, and the use of radiographic images.^3^ These techniques, even when combined, can easily lead to subjective errors. Although other methods exist, they are not mature for clinical use, being either too time-consuming or lacking the required sensitivity and selectivity.^3^ Since healthcare budgets are limited, this diverts resources from intensive treatment to those patients that need it the most. As a consequence, oral diseases rank among the top five most expensive conditions to treat, as reported by the World Health Organization (WHO).^4^ Periodontitis has also been linked to an increased risk of systemic diseases such as cardiovascular diseases^5^ and Alzheimer’s disease.^6^ Therefore, the development of methods allowing early diagnosis and treatment will not only impact oral disease management and health economics but may also be open for the use of oral biomarkers for systemic disease diagnostics.

The disease stems from poor dental hygiene and the formation of plaque, a bacterial biofilm, on teeth. Bacteria present in this biofilm are typically more protected and resistant against antimicrobials than those that are found dispersed in the oral microbiome.^2,7^*Porphyromonas gingivalis* is one such bacteria and one of the main periodontal disease pathogens.^3^ After prolonged poor dental hygiene and due to the influence of environmental factors, such as increased levels of exudate from the gingival sulcus due to inflammation, these bacteria secrete an excess of gingipains, a class of highly active proteases with the role of degrading tissue and plasma proteins to provide nutrients for bacterial growth. This triggers further recruitment of inflammatory response units such as cytokines, chemokines, and matrix metalloproteases.^8^ The inflammatory response then leads to destruction of soft tissues and alveolar bone tissues, which may lead to tooth loss if left untreated.^9,10^

Two types of gingipain proteases are produced by *P. gingivalis*, Rgp and Kgp, differing in their cleavage site preference with Rgp cleaving preferably Arg-Xaa bonds, whereas Kgp digests with preference for Lys-Xaa sites. These trypsin-like cysteine proteases are responsible for the majority of the proteolytic activity of *P. gingivalis*. Rgp exists in two forms, RgpA and RgpB. RgpA and Kgp contain both a proteolytic and an adhesion domain, whereas RgpB lacks the latter.^14^ The proteases are primarily situated in the extracellular region of the outer membrane of the bacteria but are also excreted in soluble or a vesicle-bound form (RgpB and Kgp).^10,11^ In this excreted form, the gingipains are negatively charged ca. 50 kDa proteins with isoelectric points of ca. 5.^12^

**Figure 1:**
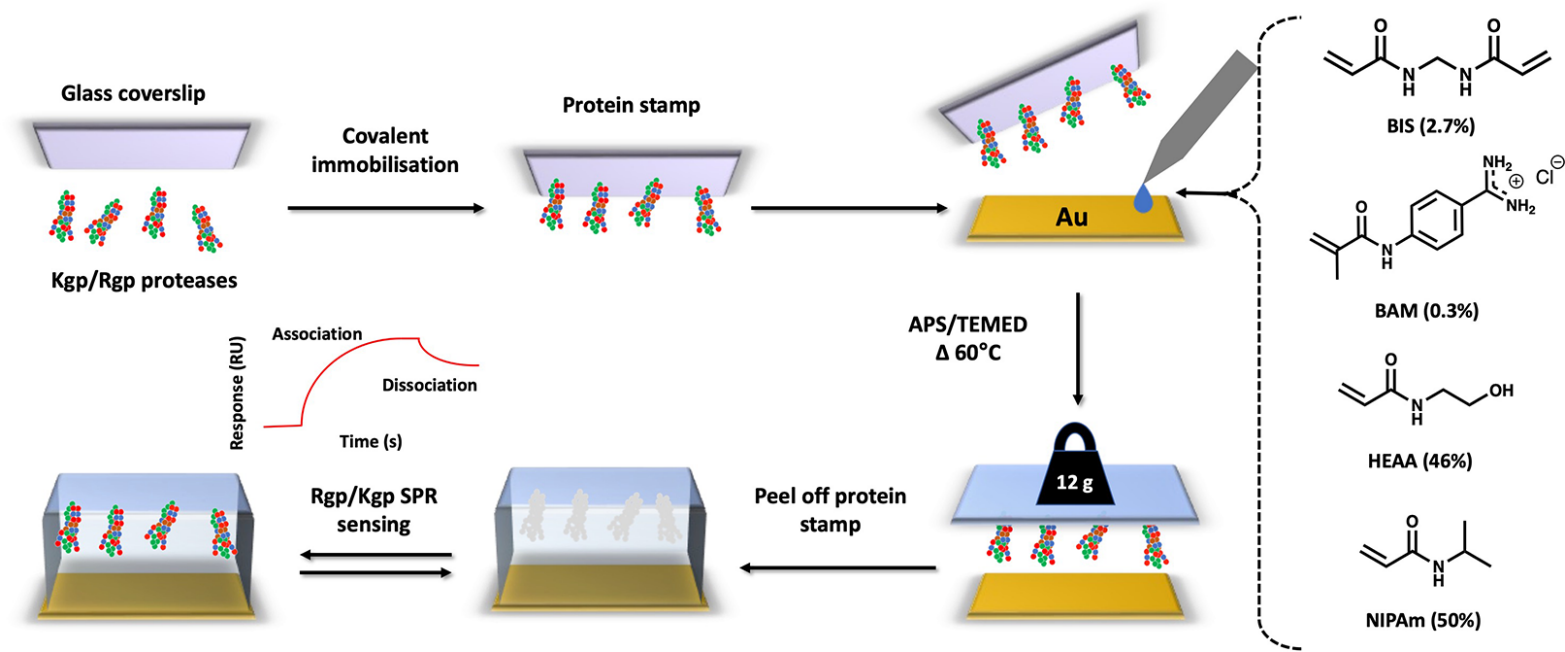
Key steps in the preparation of a μ-contact imprinted polymer film on an SPR sensor-chip. Lys-specific and Arg-specific gingipain proteases Kgp and Rgp are covalently coupled to glass microscope coverslips. A dilute solution of the indicated monomers was dropped onto an SPR sensor-chip. The protein stamp was then placed on top of the chip followed by thermal curing of the polymer overnight. After curing, the glass coverslip is removed, and the resulting MIP sensor chip is used to monitor selective binding of proteins in real time.

Previously, we reported on a sensitive nanoparticle-based nanoplasmonic biosensor for the detection of the proteolytic activity of gingipains.^13^ The sensor showed a limit of detection below gingipain concentrations detected in severe chronic periodontitis patients (∼50 μg/mL) but could not discriminate between the gingipain subtypes. To enhance the diagnostic precision, we report here on a complementary subtype selective tool capable of selectively reporting the level of gingipains. This is based on a combination of polymer-based microcontact imprinting and surface plasmon resonance (SPR) technology (Figure 1).^14,15^ In the microcontact imprinting technique, a molecularly imprinted polymer (MIP) is prepared in situ directly on the surface of the sensor by bringing a protein-modified stamp in contact with a photo- or thermally curable monomer mixture. Removing the stamp postcuring leaves behind protein recognitive sites with complementary shapes and functionalities.^16,17^ We, here, used RgpB and Kgp as protein templates to produce microcontact imprinted polymer films on gold-modified SPR sensor chips. The sensors were characterized by multicycle kinetic analysis for their binding affinity, selectivity, and sensitivity for quantifications of the targets in cell culture supernatants. Through a combination of these affinity-based sensors with our previously reported protease activity sensors, we hope to offer more detailed insight into the dysbiosis status of the subgingival biofilms.

## Materials and Methods

### Materials

Sodium hydroxide pellets (NaOH, ≥98%), (3-aminopropyl)triethoxysilane (APTES, 99%), glutaraldehyde (50% w/v in H_2_O), tyramine (99%), acryloyl chloride (>97%), triethylamine (>99.5%), hydrochloric acid (HCl, 37%), sodium phosphate monobasic monohydrate (NaH_2_PO_4_·H_2_O, ≥98%), *N*,*N*,*N*′,*N*′-tetramethylethylenediamine (TEMED, 99%), ammonium persulphate (APS, ≥98%), *N*,*N*′-methylene bis(acrylamide) (BIS, 99%), and *N*-isopropyl acrylamide (NIPAm, 97%) were purchased from Sigma Aldrich and used without further purification. *N*-(Hydroxyethyl)acrylamide (HEAA, 97%) was also purchased from Sigma Aldrich and was further diluted in H_2_O by 50%. Ethanol (absolute) and toluene (≥98%) were purchased from VWR chemicals, and sodium phosphate dibasic dihydrate (Na_2_HPO_4_·2 H_2_O, ≥98%) was purchased from Fluka Analytical. The functional monomer N-methacryloyl-4-aminobenzamidine hydrochloride (benzamidine monomer, BAM) was prepared in house, according to the procedure described in the study of Pan et al.^18^ N-His tagged Kgp (56.6 kDa) and RgpB (43.3 kDa) recombinant proteins were purchased from MyBioSource (USA, see the Supporting Information), and the competitive proteins for the selectivity analysis (chymotrypsin, trypsin, and human serum albumin (HSA)) were all purchased from Sigma Aldrich. All proteins were used without purification. Bacterial culture supernatant samples were provided by the Faculty of Odontology at Malmö university.

### Preparation of Protein Stamps

The surface of microscope cover glasses (22 × 22 mm, thickness 0.15 mm, VWR, Germany, product# 631-1570) was cleaned and activated by immersing them in 1 M HCl, milliQ water, 1 M NaOH, and a 1:1 mixture of milliQ water and ethanol for 10 min each step in a standard ultrasonic cleaning bath. At the end of this step, the cover glasses were rinsed with ethanol and dried with N_2_. Next, amino groups were introduced onto the surface by incubating the cover glasses in a 10% (v/v) mixture of APTES in ethanol at room temperature for two hours. To remove unreacted APTES, they were washed with ultrapure water and subsequently dried with N_2_. Thereafter, the cover glasses were immersed and incubated at room temperature for 18 h in a solution of glutaraldehyde (5% v/v) in sodium phosphate buffer (PB; 25 mM, pH 7.4). Excess glutaraldehyde was washed away with PB and dried under N_2_ flow. Finally, Rgp and Kgp recombinant proteins were immobilized onto the glass coverslips by incubating them in a 0.1 mg/mL protein solution in PB overnight at 4 °C. Before use, they were rinsed with PB and dried under N_2_ flow.

### SPR Sensor Chip Modifications

Bare planar gold SPR chips (Xantec, 9 × 9 × 0.3 mm) were initially rinsed with ethanol, dried under N_2_ flow, and placed in a plasma cleaner (high intensity) for 2 min. Cyclic voltammetry of a tyramine solution (10 mM) in a 3:1 10 mM phosphate buffered saline (pH 7.4) and ethanol was used to deposit a layer of tyramine onto the gold substrate. The cycle was repeated 15 times over a potential range between 0 and 1.5 mV, with a scan rate of 100 mV/s. After deposition of tyramine, the SPR chips were rinsed with milliQ water and dried under N_2_ flow. In the next step, they were incubated overnight and at room temperature in a 30 mM solution of acryloyl chloride and triethylamine in toluene.

### Preparation of Imprinted Polymers through the Microcontact Imprinting Approach

A monomer solution containing NIPAm (85.6 mg; 760 μmol), BIS (6.2 mg; 40.2 μmol), HEAA (144 μL; 77 mg; 700 μmol), BAM (1 mg, 4.4 μmol), and TEMED (20 μL, 10% v/v in PB) was combined in a small high-performance liquid chromatography (HPLC) vial with 960 μL of PB (25 mM, pH 7.4). This mixture was purged with N_2_ for 30 min at room temperature, after which APS (20 μL, 5% w/v in PB) was added. The solution was rapidly mixed and 1 μL was immediately dropped onto the modified SPR chip. The protein stamp was then brought into contact with the pre-polymerization solution by placing it on the modified SPR chip, followed by thermal curing of the polymer overnight at room temperature. To reduce film thickness variations, an extra weight (*m* = 12.5 g) was applied and kept on the stamp throughout polymerization. Thereafter, the SPR chip with the protein stamp was hydrated by immersing it in milliQ water for 2 h and then removed. Nonimprinted polymer (NIP) films were prepared by the same procedure using as stamp a bare HCl-cleaned microscope glass coverslip.

### Fourier Transform Infrared Spectroscopy

A Nicolet 6400, equipped with a liquid-nitrogen-cooled MCT-A detector, was used to perform measurements on modified SPR chips. The *smartSAGA* accessory operating at an angle of incidence of 80° was used to collect the data at resolution 4 (data spacing 1.928 cm^–1^), and the resulting spectra were the sum of a total of 250 scans. Before and during the measurements, the instrument was purged continuously with dry compressed air. A cleaned and unmodified gold SPR chip was used as the reference. OMNIC software was used to analyze the data and to correct for the baseline.

### Water Contact Angle Measurements

The contact angle of water droplets was measured using a Drop Shape Analyzer 100 instrument (Krüss). Three measurements per surface were taken and statistical average and standard deviation were calculated to investigate changes in hydrophilicity of the surface with each preparation step.

### Binding Affinity Studies of Imprinted Polymer Films with SPR

The SPR chip with the polymer film was docked into a Biacore 3000 instrument (Cytiva, Uppsala, Sweden), and a baseline was measured overnight in a running buffer (25 mM PB at pH 7.4, containing 0.005% Tween 20) at a flow rate of 5 μL/min. For the measurement of samples themselves, a multi-cycle measurement was performed over all four flow channels, at a flow rate of 20 μL/min. The association and dissociation times were set to 5 and 3 min, respectively. After dissociation, the surface was regenerated using a 10 mM glycine–HCl buffer (pH 2) during 5 min. Samples were prepared in 25 mM PB (pH 7.4) and diluted using the running buffer. The concentrations of the injected samples were in the range 125 nM–15 μM. For the compatibility tests with a biological sample matrix, W50-d (wild-type strain containing both Rgp and Kgp proteases) bacterial culture supernatants were collected and diluted 100× in the running buffer. Thereafter, Rgp was spiked at a concentration range of 125–500 nM to investigate matrix effects.

## Results and Discussion

### Sensor Preparation

The protein recognitive films of the sensors were prepared following a modified version of our previously described μ-contact imprinting procedure.^18,19^ The principle is outlined in Figure 1 and consists of the polymerization of selected monomers between a protein-modified glass surface and a gold electrode, among which the latter acts as an anchor of the polymer layer and the former as a stamp to form the surface-imprinted sites. Formation of high-fidelity imprinted sites relies on appropriate selection of the film components in the form of functional-, matrice-, and crosslinking monomers, the free radical initiation, solvent, and the protein stamp preparation.

Functional monomers are chosen to complement structural features of the template (Figure 1). Hence, for proteins carrying a net negative charge at neutral pH, enhanced imprinting is typically seen using an excess of positively charged functional monomers. Both Rgp and Kgp have isoelectric points below 5 which led us to use our previous protocol^19^ based on BAM as a functional monomer, NIPAM as a matrix monomer, and low levels of BIS as a crosslinking monomer with APS/TEMED as the redox initiator couple. The use of BAM exploits relatively stable amidine carboxylate interactions, its ability to inhibit arginine-specific protease activity, and is further justified in view of the numerous literature examples.^18,20^

To prepare the protein stamp, glass cover slips were modified with the protein templates (Rgp and Kgp with chymotrypsin included as a reference) following our previously reported procedure.^19^ The gold electrode was modified by electropolymerization of a poly-tyramine film followed by acryloylation. The degree of electrical insulation after each modification step was probed by cyclic voltammetry (CV) using the permeable redox couple Fe (CN)_6_^4–/3–^ as electro-active species in a contacting aqueous solution (Figure S1). As we expected,^21^ the redox peak intensity decreased upon each successive modification which reflects considerable reduced penetration of the conducting ions. A precise volume of the degassed prepolymerization solution was then deposited onto the modified gold surface, and after thermal curing of the polymer, the glass coverslip was removed freeing up the protein imprinted polymer film surface for subsequent SPR-based sensing (Figure 1).

### Sensor Characterization

Surface characterization of the bare, NIP, and protein-MIP sensors was performed by surface plasmon resonance (SPR) measurements, infrared reflection absorption spectroscopy (IRAS), and water contact angle measurements. The SPR equilibrium resonance unit (RUeq) values of all polymer-modified sensor chips were found to be ca. 50,000, which is within the measurable refractive index range and indicates that the imprinting process produces films with reproducible thickness. The composition of the films was subsequently investigated by IRAS. The IRAS spectra of the polymer films are shown in Figure S2 with the significant IRAS peaks highlighted and listed in Table S1. The poly-tyramine-modified surface thus featured a broad NH-stretch band in the high-frequency region and in the low-frequency region an aromatic C=C stretch signal at 1670 and 1618 cm^–1^, a signal at 1513 cm^–1^ assigned to the N–H bending vibration from the primary amine and a C–O–C ether stretching vibration at ca. 1220 cm^–1^. As expected, acryloylation led to weakening of the 1513 cm^–1^ band and bands assignable to amide I and II stretching vibrations. Postcuring, the imprinted sensor chips featured a number of strong vibrations, notably a broad band at 3297 cm^–1^ assignable to NH of the polymer backbone, signals at 2871 and 2928 cm^–1^ arising from symmetric and asymmetric CH stretch vibrations of the polymer scaffold, and a strong broad signal averaging at 1643 cm^–1^ arising from vibrations from polymer amidinium, amide, and unreacted double bonds. The signals at 1454 and 1536 cm^–1^ are assigned to amide II vibrations.

Water contact angle (WCA) measurements were used to confirm changes in surface wettability of the SPR chips upon each modification. As seen in Table S2, each step was accompanied by significant WCA changes in qualitative agreement with the expected changes in polarity and confirming that the intended modifications had occurred.

### SPR Characterization of Protein Affinity and Selectivity

Having demonstrated that the polymer films had been successfully grafted to the sensor chips, we commenced the evaluation of the SPR response to selected proteins. The Biacore 3000 instrument contains four flow channels that can be individually functionalized on-line, e.g., with ligands or receptors. This allows the option of leaving one channel unmodified and useful as a reference channel to correct for nonspecific binding, refractive index (RI) mismatches between running and injected buffer, and RI differences of the injected species. This is the common mode of assessing nano-sized MIPs, where each channel may contain a different MIP or non-imprinted polymer (NIP) for a more detailed characterization of the binding properties. In contrast, the contact imprinted films cover the entire sensor chip which precludes this mode of assessment. Background compensation is here restricted to blank subtractions accounting for buffer mismatches. Corrections for the analyte-specific RI effects were accounted for in the SPR evaluation software.

**Figure 2:**
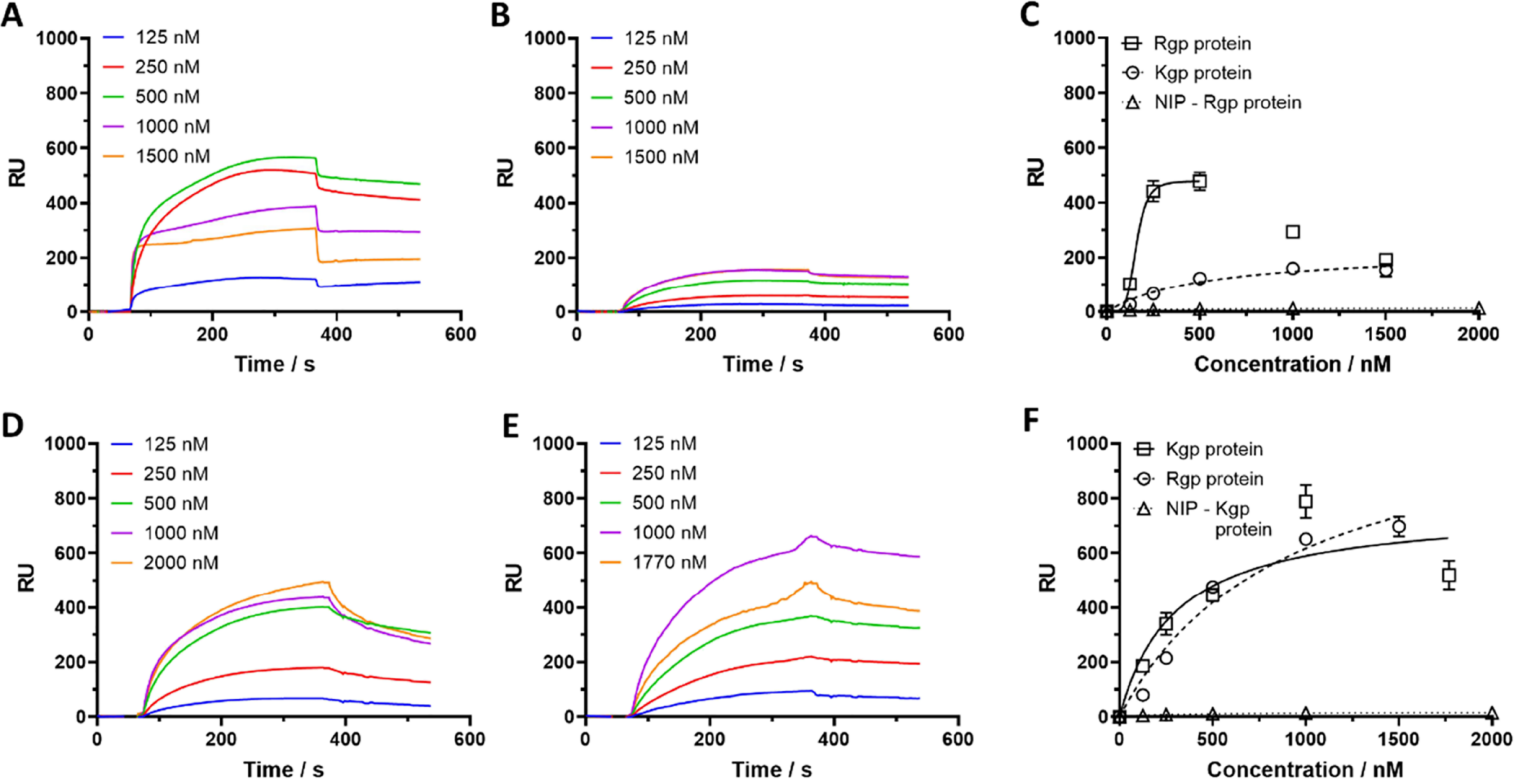
SPR sensorgrams and curves of the signal at equilibrium (RUeq) versus concentration of the injected proteins Rgp (A, D) and Kgp (B, E) using the Rgp imprinted sensor chip (A–C) and Kgp imprinted sensor chip (D–F). The relevant protein binding on the NIP surface of the signal at equilibrium is also shown (C, F). The binding curves were fitted with a Langmuir 1:1 or Hill isotherm model yielding the dissociation constants given in Table 1. Running buffer: PB (25 mM, pH 7.4, 0.005% Tween 20); regeneration buffer: Gly-HCl (10 mM, pH 2); sample injection flow rate: 20 μL/min.

The binding characteristics of each sensor chip were studied in the multi-cycle mode using PB (25 mM, pH 7.4, 0.005% Tween 20) as the running buffer and glycine–HCl (10 mM, pH 2) as the acidic regeneration buffer. Increasing concentrations (0–1500 nM) of protein standards were injected while monitoring the SPR response followed by determination of binding affinity using both equilibrium and kinetic analysis. Repeatability was verified by repeating the cycles on the same sensor chip. Figure 2 shows the sensorgrams obtained by injecting Rgp and Kgp standards onto the Rgp-, Kgp-MIP, and NIP sensor chips. First of all, we noted that the response was markedly higher on the imprinted surfaces than the nonimprinted reference, indicating the presence of imprinted sites. Comparing the two MIPs, tighter binding of the template protein appeared to occur on the Rgp-MIP. The Rgp response curves in this case (Figure 2) showed a steep RU increase in the range 0–500 nM followed by a response decrease at higher concentrations, the latter ascribed to incomplete removal of the bound protein. This was alleviated by prolonging the regeneration treatment. Injecting Kgp on this chip produced a smaller response and considerably shallower response curves. The curves were fitted to the Langmuir 1:1 model assuming a homogenous distribution of binding sites or the Hill cooperative binding model. The resulting binding affinities (*K*_d_) were estimated to be 159 and 538 nM for Rgp and Kgp, respectively, on this sensor chip (Table 1) which are within the range of protein affinities determined for other microcontact imprinted films.^14,22,23^ With respect to the Kgp-chip, imprinting appeared less effective as reflected in the smaller difference in binding affinities between these proteins. Kinetic affinity analysis was in qualitative agreement with these results but leads to markedly higher affinities for these two proteins. We ascribe this discrepancy to the slow desorption rates for these sensors which likely leads to underestimations of the rate constants. This explanation is supported by the good agreement between the two methods for faster dissociating proteins (Table 1).

**Table 1:** Characteristics of Tested Proteins and Their Corresponding Affinity Constants Calculated by Equilibrium or Kinetic Affinity Analysis

protein	molecular weight (kDa)	isoelectric point (pI)	binding affinity (*K*_d_, nM)^a^		
			Chy-MIP	Rgp-MIP	Kgp-MIP
chymotrypsin	25.0	8.75	98 ± 20 (354)	946 ± 2 (946)	1321 ± 57 (1280)
HSA	66.4	4.8	166 ± 10 (170)	1229 ± 0 (1230)	2179 ± 129 (2030)
trypsin	23.3	10.0	443 ± 35 (389)	418 ± 0 (419)	928 ± 41 (478)
Rgp	43.3	4.9–5.2	281 ± 31 (398)	159 ± 0 (3)	695 ± 65 (90)
Kgp	56.6	4.4	263 ± 6 (585)	538 ± 14 (148)	299 ± 123 (27)

a Results from kinetic affinity analysis are given in parentheses.

**Figure 3:**
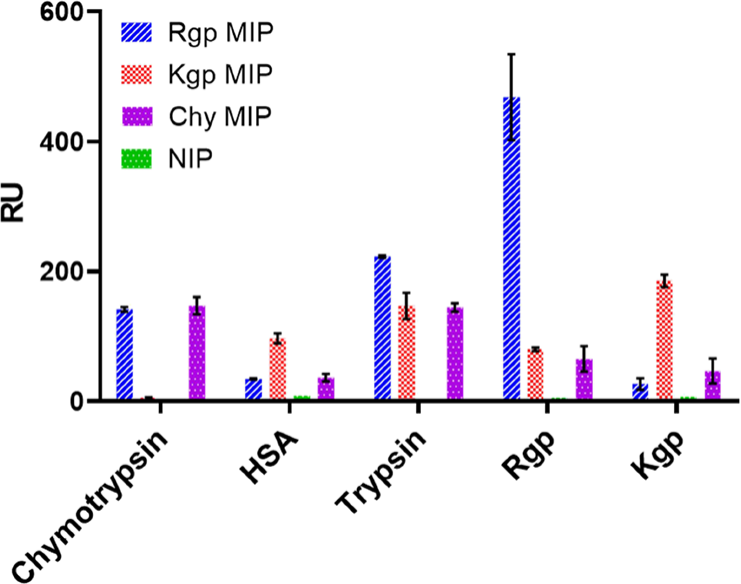
Selectivity of a polymer film imprinted with the template protein Rgp or Kgp, with a NIP film as reference against the same proteins. HSA, chymotrypsin, trypsin, Rgp and Kgp at a concentration of 125 nM in the running buffer (PB 10 mM, pH 7.4, 0.005% Tween 20) were injected separately at a flow rate of 20 μL/min. The polymer surface was regenerated in between with a glycine–HCl buffer (10 mM, pH 2). The values shown represent the response value at equilibrium, calculated by the BIA evaluation software.

We then tested the sensor’s ability to discriminate between the common proteases trypsin, chymotrypsin, and HSA as representative serum proteins. Each protein (0–15 μM) was injected over the sensor surfaces and corresponding affinities measured as mentioned above (Table 1, Figure S3). Both HSA and chymotrypsin showed low affinities (*K*_d_ > 1000 nM) whereas trypsin bound with moderate affinity, the latter ascribed to this being an arginine-specific protease prone to benzamidine inhibition (cf. the use of BAM as an affinity monomer). The selectivity of the sensors at an analyte concentration of 125 nM is shown in Figure 3. In agreement with the affinity data, all sensors displayed the highest response for the template protein with the Rgp sensor exhibiting the highest selectivity of the sensors.

The detection sensitivity, expressed as the limit of detection, was obtained from the linear range of a calibration curve covering the concentration range 125–500 nM. The limit of detection was determined by calculating 3 times the standard deviation of the intercept and converting it into a protein concentration from the slope (Figure S4). Limits of detection of 110 ± 76 and 90 ± 62 nM were determined for the Rgp and Kgp sensor corresponding to levels well below measured gingival fluid concentrations of patients with severe periodontitis.^24^

**Figure 4:**
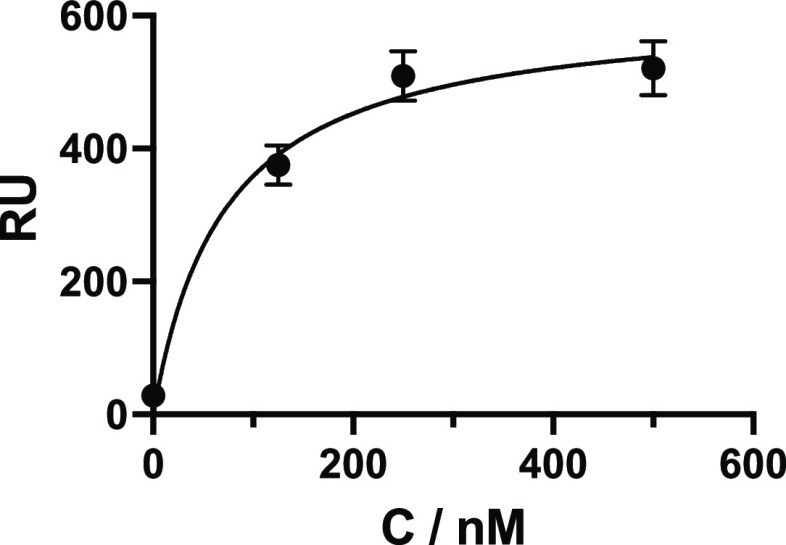
SPR signal response at equilibrium versus concentration of Rgp spiked into a diluted (100×) W50-d bacterial supernatant. Curve fitting measured data to a 1:1 binding Langmuir model resulted in *K*_d_ = 71 ± 32 nM.

Finally, to demonstrate compatibility of the sensor with a biological sample matrix, we spiked in Rgp in dilute bacterial supernatant fractions corresponding to the wild-type *P. gingivalis* strain W50-d expressing both Rgp and Kgp. As seen in Figures 4 and S5, the affinity exceeded the values obtained for the protein standards, resulting in a *K*_d_ for Rgp of 71 nM. This demonstrates that matrix effects are negligible at this dilution level and suggests the compatibility of the sensor to measure clinical sample levels of these virulence factors.

## Conclusions

Microcontact imprinted SPR sensors for the periodontitis virulence markers Rgp and Kgp were successfully prepared and characterized. Physical characterization by grazing angle infrared spectroscopy, contact angle, and SPR showed that sensors could be reproducibly prepared with respect to grafting density and composition. The binding characteristics of each sensor chip was studied by SPR in the multi-cycle mode. The Rgp sensor here displayed the highest affinity with a *K*_d_ determined by equilibrium affinity analysis of 71 nM in dilute bacterial supernatants. This sensor also displayed the highest protein selectivity and could reject Kgp as well as the competitive protease Trypsin. The Rgp sensor displayed a limit of detection of 110 nM for Rgp and proved compatible with Rgp measurements in diluted bacterial cell culture supernatants. We believe this sensor constitutes an interesting complement to our previously reported protease activity sensor for comprehensive analysis of gingipain biomarkers in periodontal disease.

## Supporting Information Available

Sequence information on the Rgp and Kgp proteinases used; experimental setup of cyclic voltammetry; cyclic voltammogram showing changes in conductivity upon modification; FTIR spectra of different SPR chip modifications and a table with peak assignments; sensorgrams of the proteins trypsin, chymotrypsin, and HSA on the Rgp and Kgp MIPs; linear regression curve of template proteins to their respective imprinted polymers; spiked Rgp sensorgram into bacterial W50-d culture supernatant; table showing the change in the water contact angle after sequential surface modifications; and response after stable overnight baseline in four flow channels for different SPR chip surfaces (PDF)
