# Supplementary material for: Microcontact-Imprinted Optical Sensors for Virulence Factors of Periodontal Disease
Source: ACS Omega. 2023 Apr 19;8(17):15259–65. doi: 10.1021/acsomega.3c00389 (PMC10157856; doi:10.1021/acsomega.3c00389)
Supplement: Supplementary file 1 — ao3c00389_si_001.pdf [file ao3c00389_si_001.pdf]

Supporting Information

Microcontact-imprinted optical sensors for virulence factors of periodontal disease

Thomas Hix-Janssens,[1] Sudhirkumar Shinde,[2] Rahma Abouhany,[1] Julia Davies,[2] Jessica Neilands,[2] Gunnel Svensäter,[2] Börje Sellergren[1]*

1) Department of Biomedical Science, Faculty of Health and Society, Malmö University, 205 06 Malmö, Sweden
2) Section for Oral Biology and Pathology, Faculty of Odontology, Malmö University, 205 06 Malmö, Sweden,

Materials and Methods

Rgp and Kgp recombinant proteins

RgpB and Kgp were purchased from MyBioSource (USA) as *E. coli* host-expressed proteins containing an N-terminal 6x His-SUMO-tag and the partial sequences of the native proteinase. The sequence of RgpB is shown below. It consists of 244 amino acids, with a reported pI of 4.89 and a molecular weight of 43.3 kDa. The total number of negatively charged residues (Asp and Glu) is 37, and the total number of positively charged residues (Arg and Lys) is 23.

YTPVEEKENG RMIVIVPKKY EEDIEDFVDW KNQRGLRTEV KVAEDIASPV TANAIQQFVK QEYEKEGNDL TYVLLVGDHK DIPAKITPGI KSDQVYGQIV GNDHYNEVFI GRFSCESKED LKTQIDRTIH YERNITTEDK WLGQALCIAS AEGGPSADNG ESDIQHENII ANLLTQYGYT KIIKCYDPGV TPKNIIDAFN GGISLANYTG HGSETAWGTS HFGTTHVKQL TNSNQLPFIF DVAC

Kgp consists of 366 amino acids, with a reported pI of 5.35 and a molecular weight of 56.6 kDa. The total number of negatively charged residues (Asp and Glu) is 39, and the total number of positively charged residues (Arg and Lys) is 28.

DVYTDHGDLY NTPVRMLVVA GAKFKEALKP WLTWKAQKGF YLDVHYTDEA EVGTTNASIK AFIHKKYNDG LAASAAPVFL ALVGDTDVIS GEKGKKTKKV TDLYYSAVDG DYFPEMYTFR MSASSPEELT NIIDKVLMYE KATMPDKSYL EKALLIAGAD SYWNPKIGQQ TIKYAVQYYY NQDHGYTDVY SYPKAPYTGC YSHLNTGVGF ANYTAHGSET SWADPSLTAT QVKALTNKDK YFLAIGNCCV TAQFDYPQPC FGEVMTRVKE KGAYAYIGSS PNSYWGEDYY WSVGANAVFG VQPTFEGTSM GSYDATFLED SYNTVNSIMW AGNLAATHAG NIGNITHIGA HYYWEAYHVL GDGSVM

Surface characterization of electrodes by cyclic voltammetry (CV)

CV experiments were carried out using a IviumStat (Ivium Technologies, Eindhoven, The Netherlands). Instrument operation and data acquisition were controlled using IviumSoft. All electrochemical measurements were conducted in a standard three electrode cell fitted with a platinum wire and a commercial Ag/AgCl electrode as the counter and the reference electrodes, respectively. Modified sensor was used as the working electrode. A solution of 0.1 M KCl containing 0.1 M potassium ferricyanide was used as the electrolyte solution. CV was performed by sweeping the potential between - 0.5 and 0.8 V at a sweep rate of 0.1 V s^{-1}. CV was used to evaluate the degree of insulation of the electrode surface after each step of electrode modification.

Supporting Figures

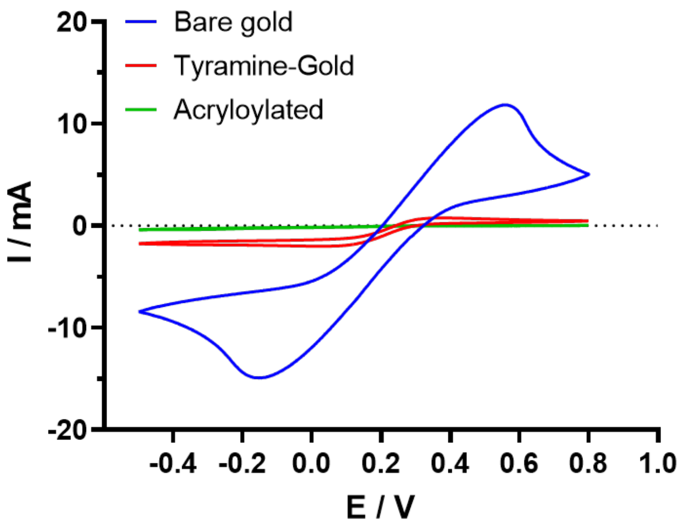

Figure S1. Cyclic voltammograms showing the conductivity of the bare gold substrate (blue curve), a substrate that has been modified with tyramine (red curve) followed by acryloyl chloride (green curve). The electrolyte solution used is the permeable redox couple Fe $(CN)_6^{4-/3-}$. Potential range used was between -0.5 V and 0.8 V, with a sweep rate of 100 mV/s.

A

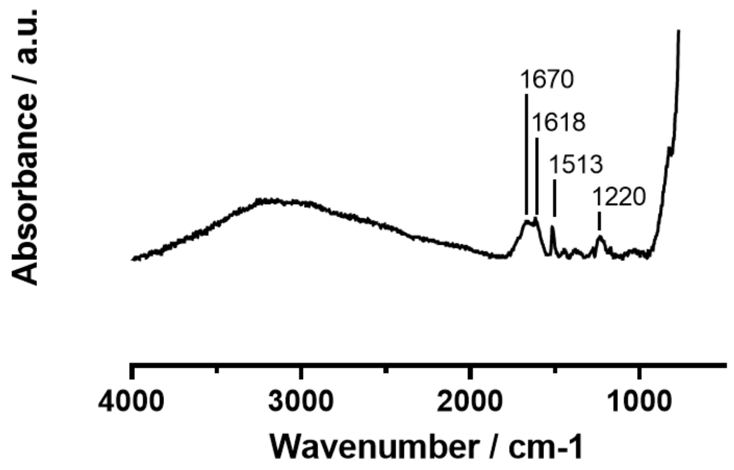

Absorbance / a.u.
1670
1618
1513
1220
4000
3000
2000
1000
Wavenumber / cm-1

B

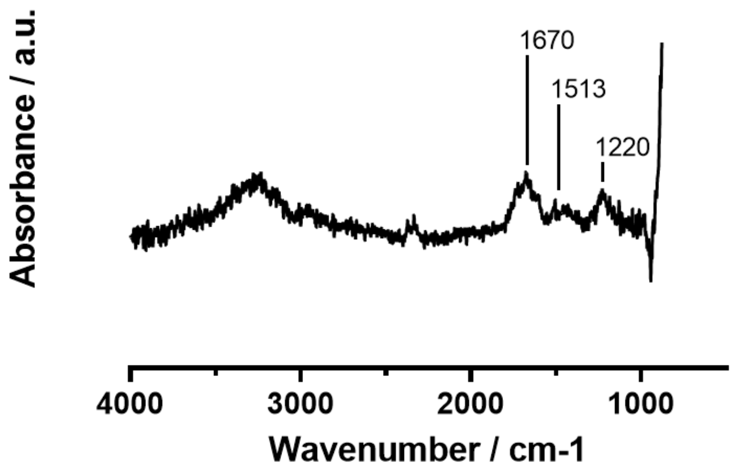

Absorbance / a.u.
1670
1513
1220
4000
3000
2000
1000
Wavenumber / cm-1

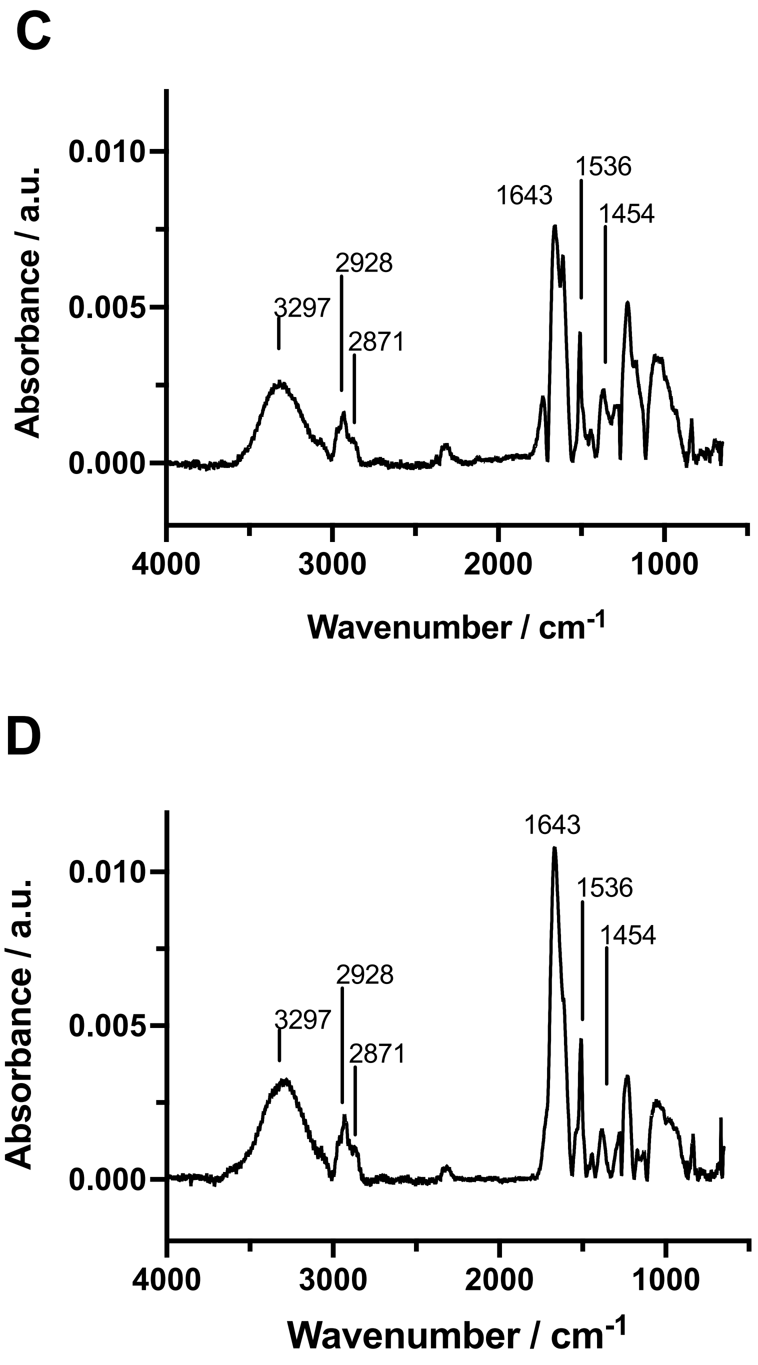

Figure S2. IRAS spectra measured of a gold SPR chip consecutively modified with (A) polytyramine, (B) acryloyl chloride, (C) a Kgp protein-imprinted MIP film and (D) an Rgp protein-imprinted MIP film. Resolution 4 (data spacing 1.928 cm^{-1}), spectra are sum of total of 250 scans.

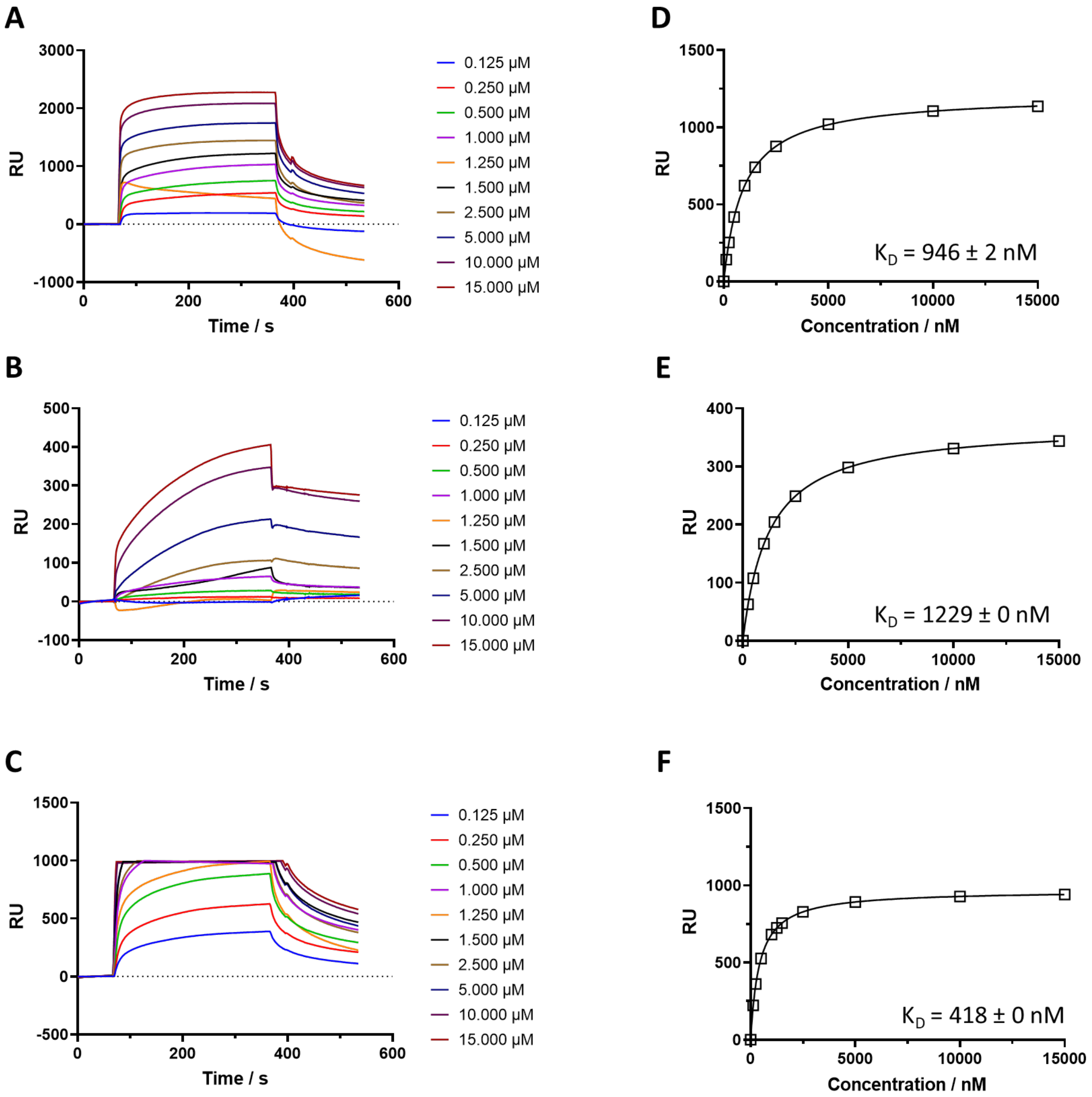

Figure S3a. SPR-sensorgrams (A-C) and curves of the signal at equilibrium (RUeq) versus concentration of the injected proteins (D-F) using the Rgp imprinted sensor chip. The latter were chymotrypsin (A,D), HSA (B,E) and trypsin (C,F). The apparent binding curves in F-J were fitted with a Langmuir 1:1 isotherm model yielding the dissociation constants given in the figure and in Table 1. Running buffer: phosphate buffer (25 mM, pH 7.4, 0.005% Tween 20); regeneration buffer: Gly-HCl (10 mM, pH 2); sample injection flow rate: 20 µL/min.

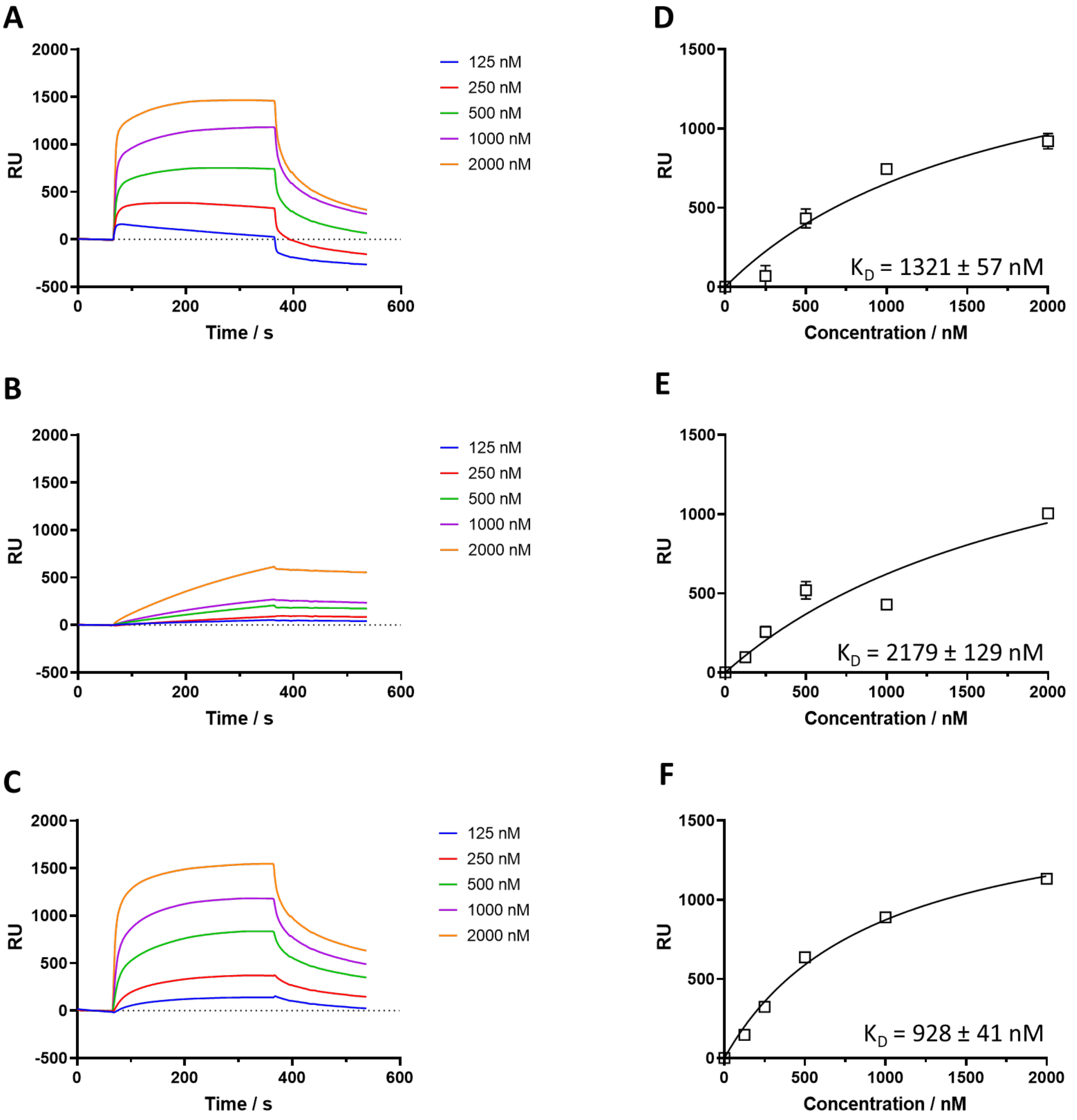

Figure S3b. SPR-sensorgrams (A-C) and curves of the signal at equilibrium (RUeq) versus concentration of the injected proteins (D-F) using the Kgp imprinted sensor chip. The latter were chymotrypsin (A,D), HSA (B,E) and trypsin (C,F). The apparent binding curves in F-J were fitted with a Langmuir 1:1 isotherm model yielding the dissociation constants given in the figure and in Table 1. Running buffer: phosphate buffer (25 mM, pH 7.4, 0.005% Tween 20); regeneration buffer: Gly-HCl (10 mM, pH 2); sample injection flow rate: 20 µL/min.

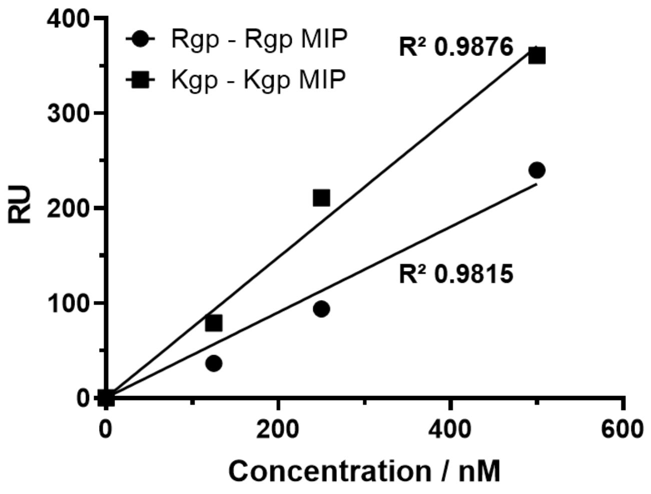

Figure S4. Linear regression curve based on the response values at the onset of desorption of both template proteins from their respective imprinted polymers.

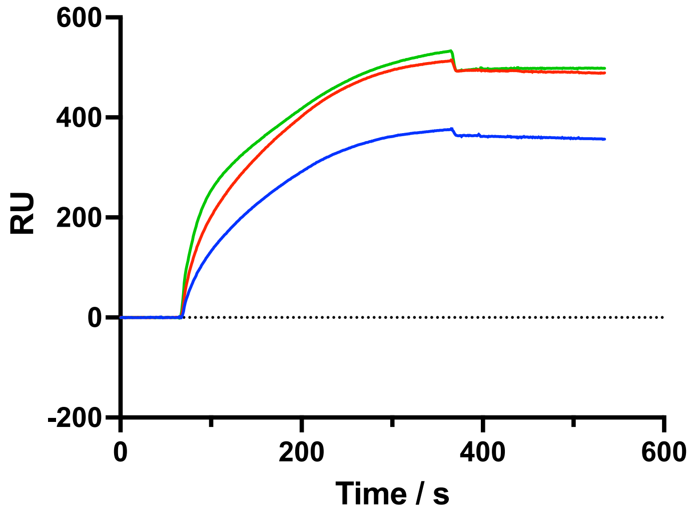

Figure S5. SPR-sensorgrams of Rgp at 125 nM (blue curve), 250 nM (red curve) and 500 nM (green curve) using the Rgp imprinted polymer sensor chip. The protein was spiked into a diluted (100x) W50-d bacterial supernatant. Running buffer: phosphate buffer (25 mM, pH 7.4, 0.005% Tween 20); regeneration buffer: Gly-HCl (10 mM, pH 2); sample injection flow rate: 20 μL/min.

Supporting Tables

Table S1. Positions and mode assignments of the IR bands of the spectra in Fig. S2

Mode assignment	Wavenumber (cm^{-1})
NH_2, N-H stretch; -OH, O-H stretch<br>CONH, N-H stretch (trans)	3230-3550
CH_2, C–H stretch	2871, 2928
Amidinium, N-C=N stretch (asym), CONH, C=O stretch	1670
C=C stretch (unreacted acryl)<br>Aromatic C=C stretch (‖1,4 axis)	1618, 1640
N-H deformation	1536
C-H deformation	1454
C-O-C stretch (p-tyramine)	1220

a) The resolution was 4 (data spacing 1.928 cm^{-1}) and the spectra the sum of 250 scans

Table S2. Water contact angles after each step of the successive surface modifications of gold SPR chips. The contact angles given are averages of three measurements.

Surface	Water contact angle (°)
Glass microscope coverslip (MC)	41.7 ± 3.4
NaOH activated MC	14.8 ± 2.9
APTES modified MC	74.7 ± 3.8
Glutaraldehyde modified MC	44.0 ± 1.8
Rgp modified MC	50.4 ± 1.2
Kgp modified MC	48.1 ± 4.7
Unmodified SPR chip	95.3 ± 1.9
Poly-tyramine modified SPR chip	76.9 ± 1.9
Acryloylated SPR chip	64.5 ± 1.9
Imprinted polymer film	61.8 ± 3.9

Table S3. Initial response values of the polymer-modified SPR chips after overnight equilibration in potassium phosphate buffer (25 mM, pH 7.4, 0.005% Tween 20) as running buffer at a flow rate of 5 μL/min.

	Initial RU value				
	Bare gold	Chymotrypsin MIP	Rgp MIP	Kgp MIP	NIP
Flow channel 1	18300	46315	50265	51925	67370
Flow channel 2	18400	44330	49440	50830	43805
Flow channel 3	18445	49750	49180	50075	43165
Flow channel 4	18440	57520	48725	51105	42515
